# Whose data set is it anyway? Sharing raw data from randomized trials

**DOI:** 10.1186/1745-6215-7-15

**Published:** 2006-05-16

**Authors:** Andrew J Vickers

**Affiliations:** 1 Departments of Epidemiology and Biostatistics, Medicine, Urology, Memorial Sloan-Kettering Cancer Center, NY, USA

## Abstract

**Background:**

Sharing of raw research data is common in many areas of medical research, genomics being perhaps the most well-known example. In the clinical trial community investigators routinely refuse to share raw data from a randomized trial without giving a reason.

**Discussion:**

Data sharing benefits numerous research-related activities: reproducing analyses; testing secondary hypotheses; developing and evaluating novel statistical methods; teaching; aiding design of future trials; meta-analysis; and, possibly, preventing error, fraud and selective reporting. Clinical trialists, however, sometimes appear overly concerned with being scooped and with misrepresentation of their work. Both possibilities can be avoided with simple measures such as inclusion of the original trialists as co-authors on any publication resulting from data sharing. Moreover, if we treat any data set as belonging to the patients who comprise it, rather than the investigators, such concerns fall away.

**Conclusion:**

Technological developments, particularly the Internet, have made data sharing generally a trivial logistical problem. Data sharing should come to be seen as an inherent part of conducting a randomized trial, similar to the way in which we consider ethical review and publication of study results. Journals and funding bodies should insist that trialists make raw data available, for example, by publishing data on the Web. If the clinical trial community continues to fail with respect to data sharing, we will only strengthen the public perception that we do clinical trials to benefit ourselves, not our patients.

## Introduction

Investigators routinely refuse to share raw data from randomized trials without giving a reason. In this paper, I will argue that attitudes towards data sharing in the clinical trial community need to rethought, drastically. I will start by sharing some personal experiences of data sharing.

### Data sharing anecdote 1: data to help plan a phase II trial

I was asked to help design an uncontrolled Phase II trial. The idea was to treat a small cohort of patients and, if the results looked promising, then design a large, randomized trial to find out for sure if the treatment was of benefit. To know whether "results looked promising" we needed some idea of how patients would do in the absence of treatment. We found a randomized, placebo-controlled trial accruing a similar group of patients to those we planned to study. We contacted the authors asking if we could obtain part of the raw data from their trial: a list of pre-treatment and follow-up scores for the control arm. Given that the principal investigator (PI) worked at the National Institutes of Health (NIH), and that the study was federally funded, we were therefore somewhat surprised when he wrote back a short note stating: "We are not prepared to release the data at this point."

### Data sharing anecdote 2: data for a meta-analysis

I faced a problem when conducting a meta-analysis: some trial reports had given summary data for number of events per patient, others had given the proportion of patients who were event-free. I wrote to the PI of one study asking for raw data so that I could convert from event numbers to event incidence. To her credit she said that she "would love to share the data" – this was again a federally funded study at the NIH – however, she was unable to do so because "my biostatistician won't release it".

### Data sharing anecdote 3: data to test a novel statistical method

I developed a novel statistical method with a colleague at Memorial Sloan-Kettering Cancer Center (MSKCC). We needed a data set on which to test our method and thought that one of the large randomized trials conducted by the cancer co-operative groups might be appropriate. We approached an MSKCC physician who had a senior position in one of these groups. After an extended discussion, he eventually allowed us to present our ideas to the appropriate committee. This involved a subsequent 45 minute telephone conference during which we pointed out that: a) the results of our analysis had no clinical implications and were of interest purely to statisticians; b) we would stress this point in any paper; c) that the co-operative group would be sent any paper before submission and would have full veto power. After a good deal of deliberation, the committee finally relented ... to let us present a written proposal. To this we heard no reply.

### Data sharing anecdote 4: data to predict the effects of a drug

A large chemoprevention trial was published suggesting that a widely used drug could help prevent a common cancer. A colleague, who knew the investigators, pointed out that the researchers had measured a certain biomarker at baseline. We have previously shown that this biomarker could predict occurrence of the cancer. My colleague therefore proposed to the trial investigators that we collaborate on a study to determine whether the biomarker could be used to predict response to the study drug. The plan was that they would release the raw data to me, I would build a statistical model and the two research groups would jointly write a paper. Our proposal was rejected.

### Data sharing anecdote 5: data to predict the effects of surgery

A randomized trial was published showing clear benefits of a certain type of cancer surgery. As it happens, the PI was a good friend of a close colleague of mine. I suggested to my colleague that we obtain raw data from the trial to see if we could identify which patients would benefit most from surgery. This was relatively straightforward: he asked his friend, his friend said yes. But the PI failed to get approval from the co-investigators and we never received the raw data.

### Data sharing anecdote 6: a positive story

I have also had several positive experiences of data sharing: one example will suffice here. I proposed to Doug Altman, who has written many of the popular *Statistics Notes *articles for the *British Medical Journal*, that we write a *Statistics Note *on analysis of covariance (ANCOVA). He thought that this was a good idea but recommended we obtain a data set to illustrate our approach. I had become friendly with Konrad Streitberger at a conference, so I emailed him asking for raw data from a recently published study, and he sent me back an Excel spreadsheet, pretty much by return email. The *Statistics Notes *paper was published with Streitberger's data [1]. Interestingly, we were subsequently contacted by numerous statisticians who wanted to use the data for teaching purposes.

### To share or not to share: guilty until proven innocent

I was never given an explicit reason why any of my requests to obtain raw data were rejected, so I can only speculate. It might be that the investigators were afraid that I might publish something that would misrepresent their findings. If so, sharing of raw data is hardly the issue: commentaries and review articles relying purely on a trial's published results are often mistaken – indeed, one article discussing the results of a surgery trial similar to that discussed in anecdote 5 stated, wholly inaccurately, that patients had died as a result of surgery[2] – and it seems more likely that raw data would improve subsequent commentary. Moreover, in three of the four cases where we planned to publish data, trialists were either invited to be co-authors or were offered the opportunity to read, revise and, if necessary, veto papers before submission. Alternatively, it might be that the trialists did not want to be scooped by their own data. Yet suggestions for collaborative publications were rejected and, moreover, I am not aware that any of the groups we have approached have published papers addressing the questions that interested us. It might be also be argued that the disproportionate fear of being scooped suggests that investigators might be overly concerned with their careers in comparison to the value of research to patients.

Whatever the reason, it is clear that there is a burden of proof problem here: "no data sharing until there is clear and unambiguous proof that no harm will result (especially to me)", rather than, "we should share data unless we have some good reason to believe that this is not in the best interests of patients." There appeared little recognition that producers of data are part of a community with reciprocal obligations, or that all science builds on previous science (untold thousands of hours of brilliant scientific work were required just to allow me to type this paper on my laptop). Moreover, there did not seem to be any appreciation that sharing of raw data may lead to techniques or findings or further research that could help alleviate human distress (appendix 1). The general discourse was, instead, "this is my data set, why should I let you have it?"

Kirwan has undertaken a more systematic study of attitudes about data sharing by surveying pharmaceutical researchers [3]. In line with my somewhat frustrating experiences, he reports that about three out of four researchers, as well as an industry group, were opposed to making raw data available from trials after publication. What is of particular interest is that Kirwan documents the reasons given by researchers against data sharing and provides a convincing riposte to each. For example, the most common argument given against data sharing was that analytical methods are pre-specified in study protocols and it is inappropriate to use other methods; in particular, an investigator analyzing shared data may engage in "data-dredging" or choose invalid forms of analysis. Kirwan argues that it is for the scientific community as a whole, not for individual trialists, to judge the suitability of any reanalysis. Note also that in the data sharing anecdotes above, the analyses I planned were entirely incidental to the main purposes of a trial and hence could not possibly have been pre-specified in the study protocol. Yet they hardly seem like data-dredging. Kirwan reports and rejects several additional arguments such as "difficulty of extracting data relating to a particular publication" (this must have been done for the data to have been analyzed); costs of administering a database (a concern for the appropriate website, not the trialists) and "an alternative analysis may itself be commercially important". This last point is particular interesting: Kirwan argues that if the value of an alternative analysis is recognized by the pharmaceutical company, it should be conducted before publication of the study results, if not, "then all the more reason for ensuring that others in the field are in a position to identify the need for and conduct the analysis".

### Whose data set is it anyway?

In 2004, I published as PI a large study of acupuncture for the treatment of chronic headache disorders [4]. At my behest, 401 patients completed numerous questionnaires over the course of a year, sometimes completing pain scores four times a day for weeks at a time. Is this data set mine? Or does it really belong to the patients in the trial, and do I act merely as custodian? Several of the trials described in the anecdotes above were literally life and death: each of the steps on the survival curve was someone's daughter, someone's father, someone's wife. And yet details of that death suddenly became a publicly-funded researcher's private property.

In the USA at least, the data *legally *belong to trialists on the grounds that it requires work to create knowledge from data. But science, particularly medical science, is essentially an enterprise conducted for moral reasons. We need to do not just what is legal but what is right. As such, we must take into account the probable wishes of the patients who give us their blood, fill in our questionnaires and die on our trials. It is difficult to believe that any patient on my trial, who completed complex questionnaires so diligently over such a long period of time, would really have wanted me to keep the data for myself rather than share it with others for the benefit of medical science in general.

### How to share data

In many cases, sharing raw data is very straightforward, here I'll do it right now: click the link for Additional File 1 to see a full data set from the acupuncture for headache trial. It did not take me particularly long to create this file. I needed to have a clean, well-annotated data set anyway in order to conduct the statistical analyses for the trial publication and so all that was required was to delete some extraneous variables, convert to Microsoft Excel, and write a few comments describing the data set. These comments could be very short as I could refer to the published trial for experimental details.

There are certain responsibilities I have as the producer of the data. First, I have to make sure my data set is clean, accurate and well-annotated, though this is good statistical practice anyway. Second, I have to make sure that I have protected patient confidentiality. This is not just a matter of deleting names and addresses, as identity can conceivably be constructed from other data, such as date of death, diagnosis and zip code (see published guidelines for help [5]). By the same token, however, anonymizing or "de-identifying" data is not particularly time consuming. Third, I have the responsibility of deciding the appropriate level of detail for a data set. For example, in the acupuncture trial, patients were given the SF-36 quality of life questionnaire on three occasions. This instrument consists of 36 questions that are summarized in 9 domains. In creating the data set, I decided not to give the responses to each question as this would require over 100 variables, rather I give the domain scores at each follow-up time.

A researcher wishing to use raw data from a randomized trial also has responsibilities. The most obvious is that the source of the data must be cited. This is comparable to the use of reference lists in papers to cite sources of published data. Second, researchers have a responsibility to involve the producers of the data set. Trialists have a deep knowledge of the data and their expertise and advice can be essential to avoid inappropriate analyses. As a general rule, trialists should be invited to be collaborators on any non-trivial novel research resulting from their data, with full co-authorship on any resulting publication.

For more thoughts on the logistics of data sharing see the datasharing.net website [6]; Appendix 1 summarizes the benefits of sharing data from randomized trials: this builds on earlier work by Hutchon [7].

### Protecting the interests of trialists

It is not implausible that a trialist's reputation or career could be harmed by data sharing. For example, what if a researcher downloaded the data set from my acupuncture trial, conducted an inappropriate analysis and then published a paper showing how the new findings "refuted" my conclusions? Similarly, if I planned a secondary analysis of my data, but was beaten to it by another researcher, I would lose the opportunity to publish a paper, and papers are, of course, the currency of a scientific career. In appendix 2, I outline some guidelines for data sharing that serve the dual function of protecting investigators and optimizing the scientific value of trials. Appendix 2 might be seen as a first attempt to answer Eysenbach and Sa's call for a "code of conduct" for publication of raw data [8].

Two features of the guidelines are of particular interest. First, they ensure that trialists are either co-authors on any subsequent analyses for publication or are mandated a published commentary along with the new analysis. Moreover, trialists are guaranteed by the guidelines to be the first to publish an analysis: they are not required to publish or share any data which, in good faith, they plan to analyze for further papers. To give a concrete example, I am currently involved in the planning of a cancer trial in which various biomarkers will be taken at baseline. I expect that we will first publish the overall results of the trial – cancer-free survival in each group – and later submit a series of papers looking at questions such as whether the biomarkers predict either survival or response to the study intervention. I do not think there is any need to make available raw data on the biomarkers until we publish papers describing our biomarker analyses.

Nonetheless, I have little doubt that guidelines will not always be followed and, even if they are, harm may still occasionally result from publication or sharing of raw data. I think we have to accept that harm will result from any policy. The key question whether the benefits from greater sharing of raw clinical trial data outweighs these harms (in my view, this is far from a close call). We also have to consider whether restricting the free flow of scientific information to avoid rare adverse events is really what we want as a research community.

## Conclusion

The NIH currently requires that grants of $500,000 or more per year provide a "data-sharing plan" in the grant application [9]. Although this is a good start, it does not ensure that data are indeed ultimately made available. Moreover, only a fraction of randomized trials are NIH-funded to the tune of $500,000 per year. My personal solution would be to make it illegal to experiment on humans and then fail to publish raw data within an appropriate period of time. I assume there may be special circumstances in which it might be reasonable to keep data confidential – for example, during early phase development of a patented drug – and one might envisage that exceptions might be granted. Lest my proposal seem rather draconian, consider whether some of the Vioxx-related deaths might have been avoided had Merck been forced to publish raw data on individual patients.

Pending the somewhat unlikely implementation of such legislation, one starting point might be the journals: what if journals, and *Trials *would be a good starting place, insisted that researchers submitted raw data, along with the journal manuscript, for publication on the journal website? I am not the first to make such a suggestion [7,10]. Adding raw data submission to the host of other requirements (conflict of interest, suggested reviewers, copyright transfer etc.) hardly seems burdensome. Indeed, trialists would merely be following the lead of genome researchers where "all scholarly scientific journals should now require the submission of microarray data to public repositories as part of the process of publication" [11]. We could also go to the funding agencies (NIH, Medical Research Council, Wellcome) and suggest they demand publication of raw data from randomized trials, in exactly the same manner that they now require Open Access publication.

The editors of *Trials *have written that they "encourage authors to make available all (or some of) the raw data from the trials" [12] A voluntary code of this nature does not seem to work. Eysenbach and Sa report that although the *Journal of Medical Internet Research *explicitly invites authors to attach raw data, none did so during the first two years of publication [8].

So perhaps what we need above all is a fundamental change of attitudes within the clinical trial community. Data sharing is common in other areas of science, genomic research being the obvious example. I cannot believe that it is unreasonable to ask clinical trialists to share data when, say, yeast researchers do it routinely. Let's make sharing of raw data a commonplace, natural part of the clinical trials process, in the same way that we view obtaining ethical approval or publication of the trial results. If we fail to do so, we will only strengthen the public perception that we do clinical trials to benefit ourselves, not our patients.

### Postscript

If you have any personal anecdotes about sharing of data from randomized trials, please post a comment to this paper or email me at vickersa@mskcc.org. Perhaps more importantly, if you are a trialist and have some data that you do not want to share, think you are justified in this and believe your rationale is not covered in my guidelines, please let me know: I want to make sure that we develop a framework that meets everyone's needs.

## Appendix 1. Benefits of sharing raw data from randomized trials

• Analyses can be reproduced and checked by others

• Acts as an additional incentive for checking that a data set is clean and accurate

• Teaching

• Aids development and evaluation of novel statistical methods

• Allows testing of secondary hypotheses

• Aids design of future trials

• Simplifies data acquisition for meta-analysis

• May help prevent fraud and selective reporting

## Appendix 2. Suggested code of conduct for analysis of published raw data

Terminology: the "trialists" are the authors of a published report of a randomized trial; "independent investigators" are a separate group of researchers who wish to analyze the raw trial data ("new analysis")

### Code of conduct for independent investigators and journals

1. Independent investigators planning to *publish *a new analysis should contact the trialists before undertaking any analyses

2. One or more trialists should be offered a co-authorship on any resulting papers

3. If trialists disagree with the methods or conclusions of a new analysis:

a. They should not have veto power, unless this was agreed beforehand by the independent investigators

b. They should, however, be guaranteed the opportunity to write a commentary to be published alongside the new analysis

4. Journals should not publish new analyses of previously published data unless either a trialist is an author or a separate commentary from a trialist is attached

5. Published new analyses should cite the original trial

### Code of conduct for trialists

1. Trialists must ensure that the data set is clean and well annotated

2. Trialists must ensure that no individual patient could be identified from the data set

3. Trialists should be *required *to share or publish immediately only those data associated with the main analyses of a published trial

a. There is no need to share data required for planned secondary analyses (e.g. correlative studies) although trialists should in good faith restrict data sharing only for analyses that they have *concrete *plans to publish

b. Regardless of any plans for future analyses, all raw data should be published or made available for sharing no longer than five years after first publication of trial results

c. There is no need to update data (e.g. as deaths accrue in a cancer trial)

4. Trialists must share all and any data requested if analyses are not to be published (e.g. data required to aid trial design)

## Supplementary Material

Additional File 1 Data set from acupuncture headache trial Click here for file
